# Dr. Peter Pickard

**Published:** 1907-11

**Authors:** 


Dr. Peter Pickard, a graduate of the medical department of the University of Buffalo, 1859, died suddenly at his home in Mount Vernon, Ohio, October 4, 1907, aged 74 years.

